# Abnormal dendritic calcium activity and synaptic depotentiation occur early in a mouse model of Alzheimer’s disease

**DOI:** 10.1186/s13024-017-0228-2

**Published:** 2017-11-14

**Authors:** Yang Bai, Miao Li, Yanmei Zhou, Lei Ma, Qian Qiao, Wanling Hu, Wei Li, Zachary Patrick Wills, Wen-Biao Gan

**Affiliations:** 1 Drug Discovery Center, Key Laboratory of Chemical Genomics, Peking University Shenzhen Graduate School, Shenzhen, 518055 China; 2 Skirball Institute, Department of Neuroscience and Physiology, New York University School of Medicine, New York, NY 10016 USA; 3 Department of Neurobiology, University of Pittsburgh, Pittsburgh, PA 15213 USA

**Keywords:** Alzheimer’s disease, Dendritic calcium activity, Two-photon imaging, APPswe/PS1dE9, Soluble Aβ oligomers, Synaptic depotentiation

## Abstract

**Background:**

Alzheimer’s disease (AD) is characterized by amyloid deposition, tangle formation as well as synapse loss. Synaptic abnormalities occur early in the pathogenesis of AD. Identifying early synaptic abnormalities and their underlying mechanisms is likely important for the prevention and treatment of AD.

**Methods:**

We performed in vivo two-photon calcium imaging to examine the activities of somas, dendrites and dendritic spines of layer 2/3 pyramidal neurons in the primary motor cortex in the APPswe/PS1dE9 mouse model of AD and age-matched wild type control mice. We also performed calcium imaging to determine the effect of Aβ oligomers on dendritic calcium activity. In addition, structural and functional two-photon imaging were used to examine the link between abnormal dendritic calcium activity and changes in dendritic spine size in the AD mouse model.

**Results:**

We found that somatic calcium activities of layer 2/3 neurons were significantly lower in the primary motor cortex of 3-month-old APPswe/PS1dE9 mice than in wild type mice during quiet resting, but not during running on a treadmill. Notably, a significantly larger fraction of apical dendrites of layer 2/3 pyramidal neurons showed calcium transients with abnormally long duration and high peak amplitudes during treadmill running in AD mice. Administration of Aβ oligomers into the brain of wild type mice also induced abnormal dendritic calcium transients during running. Furthermore, we found that the activity and size of dendritic spines were significantly reduced on dendritic branches with abnormally prolonged dendritic calcium transients in AD mice.

**Conclusion:**

Our findings show that abnormal dendritic calcium transients and synaptic depotentiation occur before amyloid plaque formation in the motor cortex of the APPswe/PS1dE9 mouse model of AD. Dendritic calcium transients with abnormally long durations and high amplitudes could be induced by soluble Aβ oligomers and contribute to synaptic deficits in the early pathogenesis of AD.

**Electronic supplementary material:**

The online version of this article (10.1186/s13024-017-0228-2) contains supplementary material, which is available to authorized users.

## Background

Amyloid plaques, neurofibrillary tangles and synapse loss are the pathological hallmarks of Alzheimer’s disease (AD) [1, 2]. AD patients exhibit progressive cognitive impairments, memory deficits and dementia. Previous studies have shown that synapse loss occurs many years before dementia and is the best correlate of cognitive impairment in AD patients [1, 3–6]. A significant reduction in postsynaptic spine density is detected before β-amyloid deposition in Tg2576 APP and PDAPP mouse models of AD [7–9]. Furthermore, down-regulation of various synaptic proteins has been observed in both AD patients and mouse models of AD [7, 10, 11]. These various synaptic deficits may underlie memory loss [8, 12, 13], as well as changes of sensory and motor systems observed at the early stage of AD [14–16].

The mechanisms underlying synaptic abnormalities and loss in AD remain unclear. Recent studies have shown that abnormal neuronal activities occur early in hippocampal and cortical regions in AD patients as well as in animal models of AD [8, 17–19]. For example, hyperactive neurons have been observed in the hippocampal CA1 region in 1.5 month-old APP23xPS45 Tg mice [19]. It has also been reported that the number of hypoactive and hyperactive cells in layer 2/3 neocortex increase in AD mice as compared to wild type mice [20]. Given the important role of neuronal activities in regulating synaptic plasticity [21, 22], alterations in neuronal activities likely cause abnormal synaptic structure and function in AD. However, the potential link between neuronal activity and synaptic abnormalities during the pathogenesis of AD remains elusive.

Many lines of evidence strongly suggest that soluble Aβ oligomers are the most neurotoxic Aβ peptides with detrimental effects on neurons and synapses in AD [17, 23–28]. The level of Aβ peptides, but not amyloid plaques, is the earliest known marker of AD and correlates with disease severity in patients [29–33]. Exogenous Aβ application into wild type mice can induce neuronal hyperactivity [34]. Furthermore, Aβ oligomers have been shown to disrupt calcium homeostasis in neurons by interacting with NMDA receptors, reducing glutamate uptake at synapses, and regulating calcium release from ER [35–41]. Because calcium elevation is an important trigger for synaptic plasticity [42, 43], these findings suggest that soluble Aβ oligomers may cause early synaptic deficits by affecting neuronal activity and calcium homeostasis in AD.

To better understand early synaptic deficits in AD, we performed transcranial two-photon calcium imaging of layer 2/3 neuronal somas, dendrites and dendritic spines in the motor cortex of 3-month-old APPswe/PS1dE9 transgenic mice before amyloid plaque formation [44–47]. We found that a fraction of dendritic branches of layer 2/3 pyramidal neurons exhibited a significant increase in the duration and amplitude of dendritic calcium transients during treadmill running in 3-month-old AD mice as compared with age-matched wild type control mice. Exogenous application of soluble Aβ oligomers into wild type mouse brain also increased the duration and peak amplitude of dendritic calcium transients during treadmill running. Notably, dendritic spines exhibited a decrease in activity and size after abnormal long-duration dendritic calcium transients. Together, these findings suggest that abnormal dendritic calcium elevation and synaptic depotentiation represent early dendritic and synaptic deficits in the pathogenesis of AD.

## Methods

### Experimental animals

The APPswe/PS1dE9 mice were purchased from Guangdong Medical Laboratory Animal Center, China. They overexpressed the Swedish mutation of APP, together with PS1 deleted in exon 9. Three-month-old mice were used in all the experiments. Mice expressing GCaMP6s in layer 2/3 and layer 5 pyramidal neurons were generated under the Thy-1 promoter in the Gan lab. Age-matched non-transgenic littermates served as controls. Mice of both sexes were used in the experiments. All experimental protocols were conducted in accordance with the institutional guidelines.

### Administration of soluble Aβ42 oligomers or monomers

Aβ peptides were synthesized and provided by Dr. Zachary Wills from University of Pittsburgh. High performance liquid chromatography (HPLC), size exclusion chromatography (SEC) and electron microscopy (EM) were used to determine the concentration and oligomeric status of Aβ peptides as described in a recent publication [48]. Synthesized Aβ42 was dissolved in DMSO and aliquoted before freezing at −80 °C. 24 h prior to the experiment, Aβ42 was thawed and vortexed for 30 s to initiate the oligomerization and then incubated at 4 °C overnight. Before use, Aβ oligomers were diluted with artificial cerebrospinal fluid (ACSF) and used within 48 h of preparation at the concentration of 20 μM. 0.5 μl solution of Aβ42 oligomers (or Aβ42 monomers and vehicle) were slowly injected into the cortex of WT mice over 50 min. The vehicle or Aβ42 monomer without overnight incubation at 4 °C was used as control groups. To assess the global effect of Aβ42, we performed calcium imaging of neurons ~ 1 h after Aβ42 injection in the motor cortex contralateral to the injection site.

### MK801 application

For local application of the NMDA receptor antagonist, MK801 (200 μM in artificial cerebrospinal fluid (ACSF), M107, Sigma-Aldrich), a glass microelectrode with a 20-μm outer diameter was inserted through a bone flap into the superficial layer of the cortex (20–30 μm below the pial surface) with an angle of 45° toward and ~100 μm away from the imaging area. The bone flap (~50 μm in diameter) for drug delivery was made adjacent to a thinned skull window for imaging.

### Treadmill running

Mice were subjected to treadmill running with the same method as described in previous studies [43, 49]. Briefly, a custom built free-floating treadmill (101 cm × 58 cm × 44 cm) was used for motor training under a two-photon microscope. This free-floating treadmill allowed head-fixed mice to move their forelimbs freely to perform motor running tasks. To minimize motion artifact during imaging, the treadmill was constructed so that all the moving parts (motor, belt and drive shaft) were isolated from the microscope stage and the supporting air-table. Animals were positioned on a custom-made head-holder device that allowed micro-metal bars to be mounted. At the onset of a running trial, the treadmill motor driven by a DC power supply was turned on and the belt speed gradually increased from 0 cm/s to 8 cm/s within ~3 s. The speed of 8 cm/s was maintained for the rest of the trial. Each mouse was trained for 6–7 trials (5 min running and 1 min resting for each trial).

### Surgery for two-photon imaging

Surgical procedures were performed similarly as described in previous studies [43, 50]. 24 h before imaging, surgery was performed to attach a head holder and to create a thinned-skull cranial window. First, mice were deeply anesthetized with an intraperitoneal injection of pentobarbital sodium (80 mg/kg). The mouse head was shaved and the skull surface was exposed with a midline scalp incision. The periosteum tissue over the skull surface was removed without damaging the temporal and occipital muscles. A head holder composed of two parallel micro-metal bars was attached to the animal’s skull to help restrain the animal’s head and reduce motion-induced artifact during imaging. A small skull region (~0.2 mm in diameter) was located over the primary motor cortex based on stereotaxic coordinates [51] (1.0 mm posterior from bregma and 1.5 mm lateral from the midline) and marked with a pencil. A thin layer of cyanoacrylate-based glue was first applied to the top of entire skull surface, and the head holder was then mounted on top of the skull with dental acrylic cement such that the marked skull region was exposed between the two bars. Precaution was taken not to cover the marked region with dental acrylic cement.

Before imaging of dendrites and dendritic spines, a high-speed micro-drill and microsurgical blade were used to thin a circular area over the marked region to a thickness of approximately 20 μm. The thinning procedure generally took less than 5 min.

To image somas at the depth of ~250 μm below the pial surface, the skull overlying the motor cortex of interest (1 mm × 1 mm) was removed and replaced with a glass window right before two-photon imaging as described previously [43].

### Two-photon imaging of somas, dendrites and dendritic spines in the motor cortex

Genetically encoded calcium indicator GCaMP6s was used for calcium imaging in the primary motor cortex of awake, head-restrained mice. For calcium imaging of APPswe/PS1dE9 mice and wild type controls, a total amount of 0.1–0.2 μl of AAV-synapsin-GCaMP6s (AAV serotype 2/1; >2 × 10^13^ (GC/ml) titer; from the University of Pennsylvania Gene Therapy Program Vector Core) was diluted two times in ACSF and slowly injected (Picospritzer III; 20 p.s.i., 20 ms, 0.3 Hz) over 10–15 min into layer 2/3 of the motor cortex (0–1.5 mm posterior from bregma and 0–1.5 mm lateral from midline) using a glass microelectrode. Mice were prepared for calcium imaging ~18 days after virus injection.

For calcium imaging of mice injected with Aβ42 oligomers and monomers, we used wild type mice infected with AAV-synapsin-GCaMP6s, as well as transgenic mice expressing GCaMP6s in layer 2/3 and layer 5 pyramidal neurons under the Thy-1 promoter. This transgenic mouse line was generated in the Gan lab. Calcium imaging of dendrites and somas was performed in mice immediately after Aβ42 injection.

For simultaneous calcium and structural imaging, three viruses (AAV1.hSyn.Cre.WPRE.hGH, AAV1.CAG.Flex.tdTomato.WPRE.bGH and AAV1.CAG.Flex.GCaMP6s.WPRE.SV40 from the University of Pennsylvania Gene Therapy Program Vector Core) were mixed at 1:15:80 volume ratios, and ~0.1 μl of the three mixed viruses were injected into layer 2/3 of the primary motor cortex. Viruses were injected into the primary motor cortex ~18 days before calcium and dendritic spine imaging.

To image somas at the depth of ~250 μm below the pial surface, the skull overlying the motor cortex of interest (1 mm × 1 mm) was removed and replaced with a glass window right before two-photon imaging. Dendritic calcium transients and spine calcium transients were imaged at the depth of ~50 μm below the pial surface through a thinned skull window. Neuronal somas were imaged at the depth of ~250 μm below the pial surface via a glass window. The imaging window was immersed in ACSF and the head-restrained animal was placed on the stage of a two-photon laser scanning microscopy (Olympus Fluoview 1000 two-photon system equipped with MaiTai DeepSee Ti:Sapphire laser from Spectra Physics). The laser was tuned to the wavelength of 920 nm with laser power of ~ 20 mW on the tissue sample. Calcium signals were recorded at 2 Hz using a 25X objective (N.A. 1.05, 3X digital zoom). Dendritic spine structures were imaged at 0 h and 1.5 h using a 25X objective (N.A. 1.05, 3X digital zoom).

During quiet resting state, we took images for 60 s. During running, we acquired images for 5 running trials. Each running trial lasted 60 s. After the completion of each trial, the treadmill was turned off and the next trial started after a 60-s rest period.

### Image analysis

Neuronal, dendritic and spine calcium activity, indicated by GCaMP6 fluorescence changes, were analyzed post hoc using Image J software (NIH). The GCaMP6 fluorescence (F) during quiet resting and running was measured by averaging pixels within each identifiable area (Regions of interests, ROIs). Then ∆F/F_0_ was calculated as ∆F/F_0_ = (F-F_0_)/ F_0_, in which ∆F was F-F_0_ (all F values were subtracted a background fluorescence value of vessels), and F_0_ was the average of 10% minimum F values over 1 min period, representing baseline fluorescence. The threshold for determining calcium transients was calculated as three times the standard deviation (SD) of baseline fluorescence. The frequency of calcium transients was calculated as the number of calcium transients per minute on each dendrite. The duration was calculated as the total time of calcium transients with ∆F/F_0_ above the threshold. The peak amplitude was the highest value of the calcium transient. The total calcium activity was the accumulated calcium activities above the threshold per minute.

Similar to previous studies [42], back-propagating action potential-related calcium component was removed from spine calcium signals by subtracting a scaled version of the dendritic shaft signal, ΔF/F_0spine_specific_ = ΔF/F_0spine_ – 0.7 x ΔF/F_0dendrite_. We defined active spines as those with spine-specific calcium transients that crossed 3 SD of baseline fluorescence and inactive spines as those less than 3 SD.

Spine head size was measured according to previous studies [43, 49]. After background subtraction, the fluorescence intensity of tdTomato in the spine (the intensity of all pixels covering the spine in the best focal plane) was divided by the fluorescence intensity of the adjacent dendritic shaft. tdTomato fluorescence intensity of a spine was measured as follows, where Area was the number of pixels in an oval surrounding the head of the spine and mean optical density (Mean OD) was the mean brightness of pixels in that area:

The ratio of spine head diameter to adjacent dendritic shaft diameter = (Area (of spine) × Mean OD (of spine) - Area (of spine) × Mean OD (of background))/(Area (of spine) × Mean OD (of dendrite) - Area (of spine) × Mean OD (of background)). The Mean OD of both the background and the dendrite was calculated from measurements taken next to each spine, averaged for each dendrite segment.

After spine head sizes were measured on the same dendritic segment, spine size changes were calculated by comparing spine size measurement between imaging sessions.

### Statistics

All data were presented as mean ± s.e.m. The Kolmogorov-Smirnov test was used to test whether datasets were normally distributed. If so, we used a two-tailed Student’s t test to compare two groups. If not, tests for differences between groups were performed using non-parametric tests. Two Independent-samples Mann-Whitney Test and Related-samples Wilcoxon Signed Rank Test ware used to compare differences between groups whose distributions did not pass Kolmogorov-Smirnov test. Significant levels were set at *p* ≤ 0.05. All statistical analyses were performed using the IBM SPSS Statistics 23.

## Results

### Somatic calcium activities of layer 2/3 neurons are reduced in 3-month-old AD mice than in WT mice during quiet rest, but not treadmill running

**Fig. 1:**
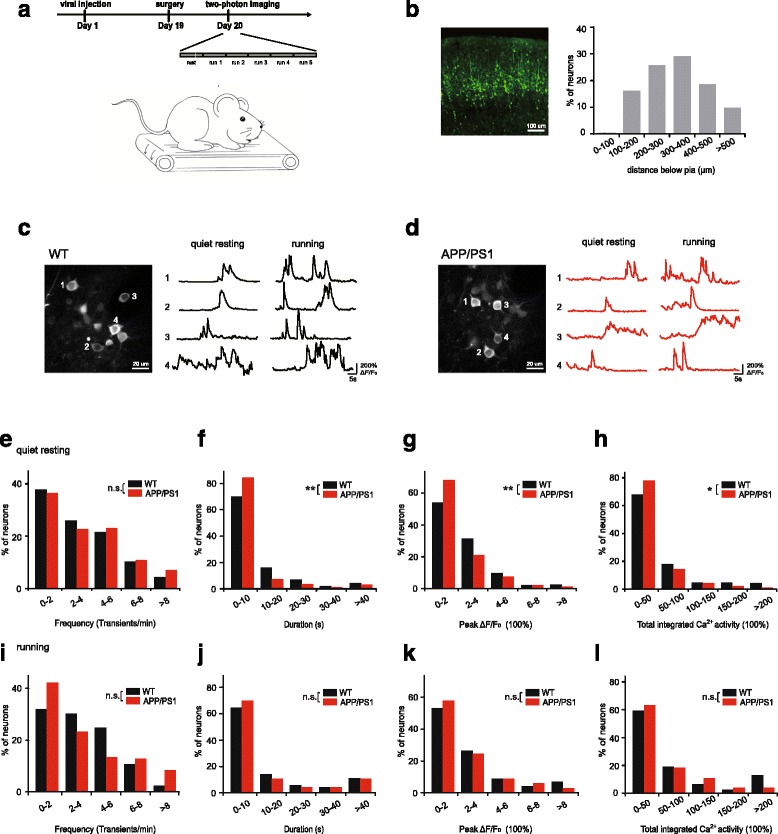
Somatic calcium activities of APP/PS1 mice are lower than WT mice during quiet resting, but not during treadmill running. **a**. Experimental design. After viral infection and surgery, layer 2/3 neurons of the motor cortex were imaged over 1 min under quite resting and over 5 treadmill running trials (60 s each trial). **b**. The confocal image of neurons labeled by AAV-syn-GCaMP6s in the mouse primary motor cortex (left); Distance distribution of GCaMP6s-expressing somas shows most of the labeled cells are in layer 2/3 (right). **c**. A representative image of somatic calcium activity in WT mice (left). Fluorescence traces of 4 different somas during quiet resting and running states (right). **d**. A representative image of somatic calcium activity in APP/PS1 mice (left). Fluorescence traces of 4 different somas during quiet resting and running states (right). **e**-**h**. Distributions of the frequency (**e**), duration (**f**), peak ΔF/F_0_ (**g**) and total integrated activity (**h**) of somatic calcium transients in WT and APP/PS1 during quiet resting (WT: 4 mice, 185 somas; APP/PS1: 5 mice, 186 somas. Mann-Whitney U Test). **i**-**l**. Distributions of the frequency (**i**), duration (**j**), peak ΔF/F_0_ (**k**) and total integrated activity (**l**) of somatic calcium transients in WT and APP/PS1 during treadmill running (WT: 4 mice, 169 somas; APP/PS1: 5 mice, 180 somas. Mann-Whitney U Test). *P < 0.05, **P < 0.01, ***P < 0.001. n.s., not significant

To investigate early neuronal deficits in the mouse model of AD, we first performed in vivo two-photon imaging of somatic calcium activities of layer 2/3 (L2/3) neurons expressing a genetically encoded calcium indicator (GCaMP6s) in the primary motor cortex (M1) of 3-month-old APPswe/PS1dE9 mice (Fig. 1a). Expression of GCaMP6s was achieved by injecting recombinant adeno-associated virus (AAV) encoding GCaMP6s under the human synapsin-1 (SYN1) promoter into the M1 region. Three weeks after viral injection, ~80% GCaMP6-expressing somas were located between 120 and 460 μm below the pia surface (Fig. 1b; 500 somas from 5 mice), indicating that GCaMP6 was expressed mostly in L2/3 neurons.

We imaged somatic calcium activities of L2/3 neurons in M1 under both resting and running conditions (Fig. 1c, d). Under the quiet resting condition, the frequency of calcium transients was comparable between AD and WT mice (Fig. 1e; WT: *n* = 185 somas from 4 mice; AD: *n* = 186 somas from 5 mice; *P* = 0.519). The duration, peak ΔF/F_0_ and integrated somatic calcium activity over 1 min were significantly lower in AD mice compared to WT mice (Fig. 1f-h, *P* < 0.05). On the other hand, when the mice were performing a treadmill running task, there was no significant difference in the frequency, duration, peak ΔF/F_0_ and integrated somatic calcium transients between AD and WT mice (Fig. 1i-l; WT: *n* = 169 somas from 4 mice. AD: *n* = 180 somas from 5 mice. *P* > 0.2). Thus, different from hyperactivity reported in the hippocampus of 1.5-month-old APP23 × PS45 mice [19], L2/3 neurons in the M1 had a lower level of somatic calcium activity under quiet resting but not during treadmill running in 3-month-old APPswe/PS1dE9 mice when compared with WT mice.

### The overall level of synaptic activities on apical dendrites of layer 2/3 pyramidal neurons is comparable between WT and AD mice

**Fig. 2:**
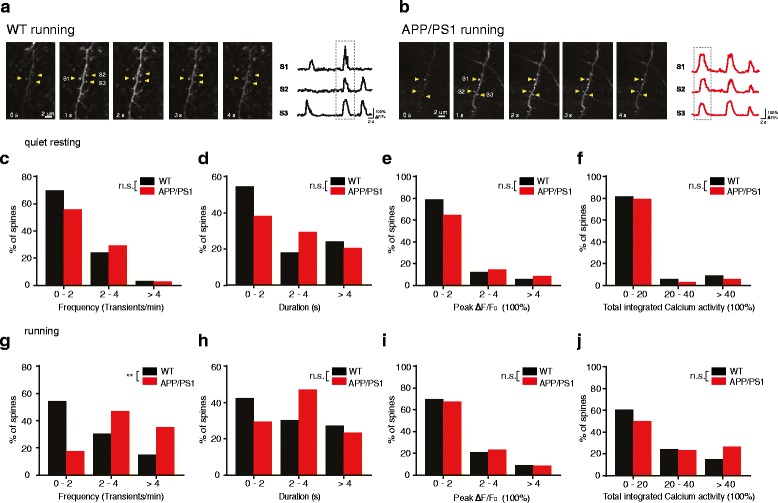
The overall synaptic activity on apical dendrites is comparable between WT and APP/PS1 mice. **a**, **b**. Time-lapse images of dendritic spine calcium transients (left) and fluorescent traces of dendritic spines expressing GCaMP6s (right) in WT (**a**) and APP/PS1 mice (**b**). Calcium transients in three spines (arrowheads point to each spine) were shown. **c**-**f**. Distributions of the frequency (**c**), duration (**d**), peak ΔF/F_0_ (**e**) and total integrated activity (**f**) of spine calcium transients in WT and APP/PS1 mice under quiet resting state (WT:4 mice, 32 spines; APP/PS1: 5 mice, 30 spines. Mann-Whitney U Test). **g**-**j**. Distributions of the frequency (**g**), duration (**h**),peak ΔF/F_0_ (**i**) and total integrated activity (**j**) of spine calcium transientsin WT and APP/PS1 mice during treadmill running (WT: 4 mice, 33 spines; APP/PS1: 5 mice, 34 spines. Mann-Whitney U Test). **P < 0.01; n.s., not significant

In addition to somatic calcium transients, we also examined calcium transients in dendritic spines on apical dendrites of layer 2/3 pyramidal neurons in WT and AD mice infected with GCaMP6s viruses (Fig. 2a, b). Under the quiet resting condition, we found no significant difference in the frequency, duration, peak ΔF/F_0_ and total integrated activity of spine calcium transients between WT and AD mice (Fig. 2c-f; WT: *n* = 4 mice, 32 spines; AD: *n* = 5 mice, 30 spines. *P* > 0.1).

When mice were subjected to treadmill running, the frequency of spine calcium transients was significantly higher in AD mice as compared to that in WT mice (Fig. 2g; WT: *n* = 33 spines from 4 mice; AD: *n* = 34 spines from 5 mice. *P* = 0.002). The duration, peak amplitude and total integrated activity of spine calcium transients were comparable between AD and WT mice (Fig. 2h - j; *P* > 0.3). Together, these results indicate that the overall level of synaptic activity is comparable between WT and AD mice under both quiet resting and running conditions.

### The duration and peak ΔF/F_0_ of dendritic calcium transients are significantly higher during running in AD mice than in WT mice

Many lines of evidence indicate that dendritic calcium spikes are critical for synaptic plasticity of excitatory neurons [52–57]. Recent studies have shown that dendritic calcium spikes in apical dendrites of layer 5 pyramidal neurons are important for dendritic spine plasticity in the motor cortex [43]. To investigate potential dendritic deficits in AD mice, we examined dendritic calcium activity of L2/3 pyramidal neurons in WT and AD mice under both quiet resting and treadmill running conditions.

**Fig. 3:**
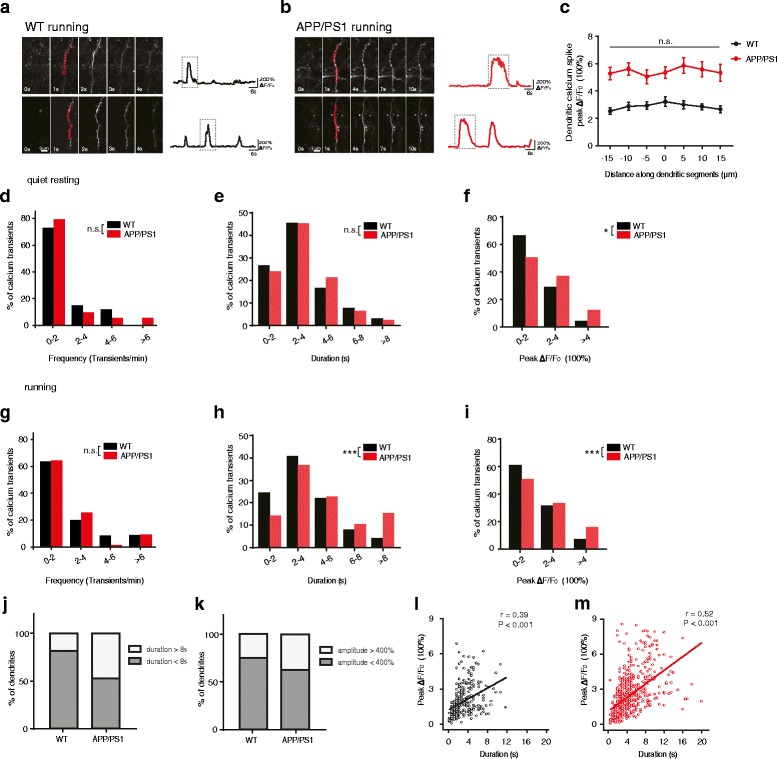
The duration and peak ΔF/F_0_ of dendritic calcium transients are higher in APP/PS1 mice than in WT mice during running. **a**-**b**. Time-lapse images of dendritic calcium transients in WT (**a**) and APP/PS1 mice (**b**) during running. Apical dendrites of layer 2/3 pyramidal neurons exhibiting large calcium transients at time point 1 s were shown in red. Their calcium fluorescence traces were shown on the right. **c**. Measurements of calcium fluorescence along long dendritic segments in the plane of imaging. Comparable fluorescent signals were observed across dendritic segments in both WT (*n* = 10) and APP/PS1 mice (*n* = 10). **d**-**f**. Distributions of the frequency (**d**), duration (**e**) and peak ΔF/F_0_ (f) of dendritic calcium transients in WT and APP/PS1 mice under quiet resting state (WT:4 mice, 90 dendritic calcium transients; APP/PS1: 5 mice, 121 dendritic calcium transients. Mann-Whitney U Test). **g**-**i**. Distributions of the frequency (**g**), duration (**h**) and peak ΔF/F_0_ (**i**) of dendritic calcium transients in WT and APP/PS1 mice during treadmill running (WT: 4 mice, 297 dendritic calcium transients; APP/PS1: 5 mice, 543 dendritic calcium transients. Mann-Whitney U Test). **j**. The percentage of dendrites with at least one prolonged dendritic calcium transient (duration >8 s) during running in WT and APP/PS1 mice. **k**. The percentage of dendrites with at least one dendritic calcium transient with large peak amplitude (amplitude >400%) during running in WT and APP/PS1 mice. **l**-**m**. The duration of individual dendritic calcium transients correlates with the peak amplitude of calcium transients in both WT (**l**) and APP/PS1 mice (**m**) during running (WT: 4 mice, 297 dendritic calcium transients; APP/PS1: 5 mice, 543 dendritic calcium transients). Data in c are represented as the mean ± s.e.m. *P < 0.05, **P < 0.01, ***P < 0.001. n.s., not significant

In the superficial layer of the motor cortex, we observed large dendritic calcium transients that occurred across long segments (> 30 μm) of apical tuft dendrites of layer 2/3 pyramidal neurons (Fig. 3a-c). The vast majority of these dendritic calcium transients lasted more than hundreds of milliseconds and had comparable ΔF/F_0_ across long stretches of dendrites, resembling NMDAR-activation-dependent dendritic calcium spikes described previously [43, 58–60] (Fig. 3c). Under the quiet resting condition, we found that the frequency and duration of such dendritic calcium transients were not significantly different between WT and AD mice (Fig. 3d, e; WT: *n* = 90 calcium transients from 4 mice; AD: *n* = 121 calcium transients from 5 mice; *P* > 0.05). However, the peak ΔF/F_0_ of dendritic calcium transients was significantly higher in AD mice than in WT mice (Fig. 3f; *P* = 0.005).

In mice running on the treadmill, we found that the frequency distribution of dendritic calcium transients was comparable between WT and AD mice (Fig. 3g; WT: *n* = 297 calcium transients from 4 mice; AD: *n* = 543 calcium transients from 5 mice. *P* = 0.774), whereas both the duration and peak amplitude distribution were significantly different (Fig. 3h, i; *P* < 0.002). Notably, a significantly larger fraction of dendritic calcium transients exhibited longer duration (> 8 s) and higher peak amplitude in AD mice than in WT mice (Fig. 3h, i).

Of all dendrites exhibiting dendritic calcium transients during a 5-min running period, ~47% and ~18% of dendrites exhibited at least one long-duration (> 8 s) dendritic calcium activity in AD mice and WT mice, respectively (Fig. 3j). In addition, 37.2% of dendrites showed at least one calcium transient with large peak amplitude (> 400%) in AD mice, higher than that (25.0%) in WT mice (Fig. 3k). There was a strong correlation between the duration and peak amplitude of dendritic calcium transients in both WT and AD, indicating that dendritic calcium transients with longer duration tended to have larger calcium peak amplitude (Fig. 3l, m; WT: *n* = 297 transients from 4 mice, *r* = 0.039, *P* < 0.001; AD: *n* = 543 transients from 5 mice, *r* = 0.052, *P* < 0.001). Together, these results indicate that even though overall dendritic spine activities were comparable between AD and WT mice (Fig. 2), a significantly larger fraction of dendrites exhibited dendritic calcium transients with abnormal long-duration and high peak amplitude in L2/3 pyramidal neurons in the M1 region of 3-month-old AD mice than in WT mice.

### Exogenous soluble Aβ42 oligomers induce dendritic calcium transients with long duration and high peak amplitude during treadmill running

**Fig. 4:**
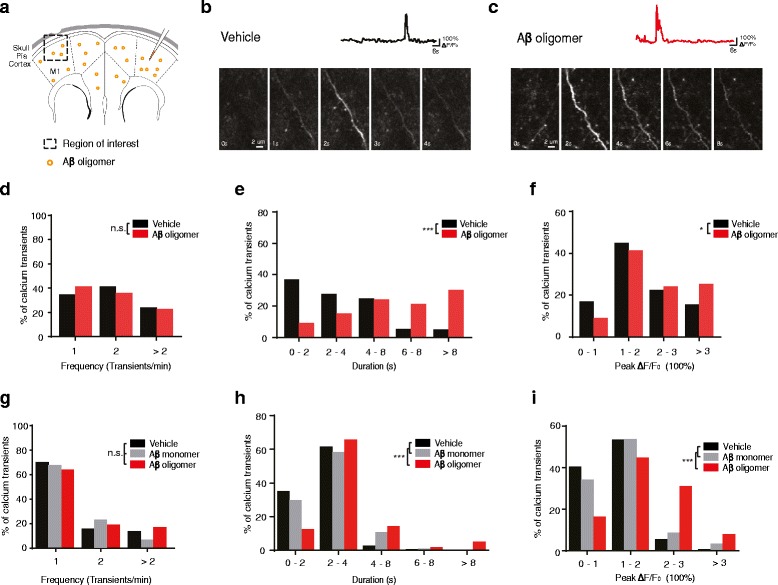
Soluble Aβ oligomer injection induces dendritic calcium transients with abnormally long duration and high peak amplitude during running. **a**. Schematic showing the injection of soluble Aβ oligomers in the brain and the imaging area contralateral to the inject site. **b**-**c**. Time-lapse images of dendritic calcium transients in vehicle (**b**) and Aβ injected mice (**c**) during running. Top, traces of calcium transient fluorescence during running. **d**-**f**. Distributions of the frequency (**d**), duration (**e**) and peak ΔF/F_0_ (**f**) of dendritic calcium transients between vehicle and Aβ oligomer-injected mice during treadmill running. Mice were infected with AAV to express GCaMP6s in layer 2/3 neurons in the motor cortex (Vehicle: 4 mice, 136 dendritic calcium transients; Aβ oligomer: 4 mice, 99 dendritic calcium transients. Mann-Whitney U Test). **g**-**i**. Distributions of the frequency (**g**), duration (**h**) and peak ΔF/F_0_ (**i**) of dendritic calcium transients in Aβ oligomer, Aβ monomer and vehicle-injected mice during treadmill running in Thy-1 GCaMP6 transgenic mice (Vehicle: 4 mice, 148 dendritic calcium transients; Aβ monomer: 4 mice, 176 dendritic calcium transients; Aβ oligomer: 4 mice, 152 dendritic calcium transients. Mann-Whitney U Test). ***P < 0.001. n.s., not significant

Many lines of evidence indicate that soluble Aβ42 oligomers can alter neuronal activity and lead to synaptic loss [61–65]. To investigate the effect of Aβ oligomers on neuronal activities in vivo, we injected Aβ oligomers into the motor cortex of wild type mice infected with AAV-hSyn-GCaMP6s virus. One hour after the injection of Aβ oligomers, we performed calcium imaging of somas, spines and apical dendrites of pyramidal neurons in the M1 contralateral to the site of Aβ injection (Fig. 4a-c). During treadmill running, we found that Aβ oligomers induced significantly higher somatic calcium activities (Additional file 1: Figure S1) but had no significant effect on the overall level of spine calcium activities of layer 2/3 pyramidal neurons (Additional file 2: Figure S2). Notably, while the frequency of dendritic calcium transients was not significantly different between vehicle and Aβ oligomer-injected mice (Fig. 4d; Vehicle: *n* = 136 calcium transients from 4 mice; Aβ42 oligomer: *n* = 99 calcium transients from 4 mice; *P* = 0.034), the injection of Aβ oligomers caused a significantly longer duration and higher peak amplitude of dendritic calcium transients as compared to vehicle injection (Fig. 4e-f; *P* < 0.05). These findings indicate that during treadmill running, Aβ oligomers cause a rapid increase in neuronal somatic calcium activities, as well as abnormally long duration of dendritic calcium transients in layer 2/3 neurons of the motor cortex.

To investigate the effects of Aβ oligomers further, we also examined dendritic calcium activities of pyramidal neurons in the motor cortex of transgenic mice expressing GCaMP6s after injecting either Aβ oligomers or Aβ monomers. During treadmill running, the frequency of dendritic calcium transients was not significantly different among Aβ oligomer, monomer and vehicle-injected mice (Fig. 4g; Vehicle: *n* = 148 dendritic calcium transients from 4 mice; Aβ monomer: *n* = 176 calcium transients from 4 mice; Aβ42 oligomer: *n* = 152 calcium transients from 4 mice. *P* > 0.3). However, administration of Aβ oligomers significantly increased the duration and peak amplitude of dendritic calcium transients when compared to control mice (Fig. 4h-i; *P* < 0.001). Taken together, these findings using mice infected with GCaMP6-expressing AAV or GCaMP6 transgenic mice suggest that exogenous soluble Aβ oligomers, but not Aβ monomers, cause abnormal dendritic calcium transients with long duration and high amplitude in mice during treadmill running.

### The prolonged dendritic calcium transients reduce the peak ΔF/F_0_ of dendritic spine calcium transients in AD mice

**Fig. 5:**
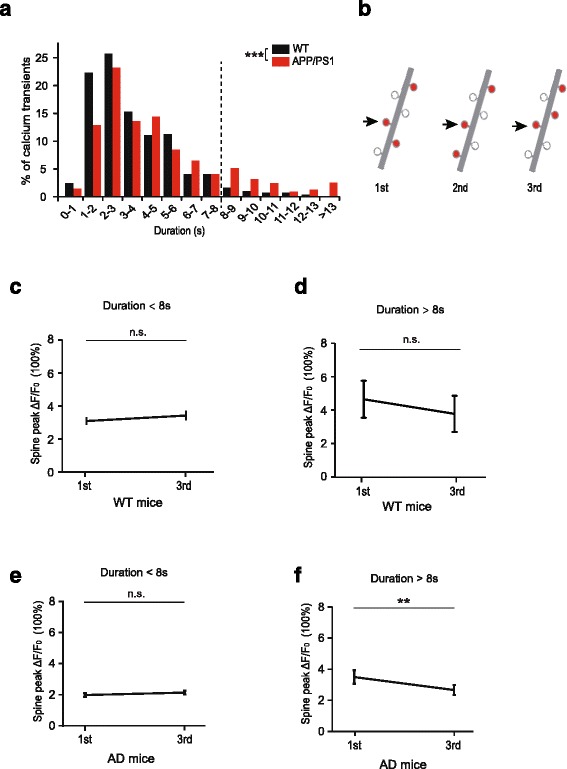
Dendritic calcium transients with abnormal long-durations reduce peak amplitudes of spine calcium transients in APP/PS1 mice. **a**. Distributions of individual dendritic calcium transients in WT and APP/PS1 mice during running (WT: 4 mice, 297 calcium transients; APP/PS1:5 mice, 543 calcium transients. Mann-Whitney U Test). **b**. Schematic showing a dendritic branch exhibiting three dendritic calcium transients (1st, 2nd and 3rd) during a running trial. The peak ΔF/F_0_ of spine calcium transients was compared between 1st and 3rd image to examine the potential effect of the 2nd dendritic calcium activity. **c-d**. Changes of spine peak ΔF/F_0_ after the 2nd dendritic calcium transient in WT mice.The groups are divided according to the duration of the 2nd dendritic calcium transient: 0–8 s (c) and >8 s (d) respectively (0–8 s, 79 spine calcium transients; > 8 s, 8 spine calcium transients). **e**-**f**. Changes of spine peak ΔF/F_0_ after the 2nd dendritic calcium transient in APP/PS1 mice.The groups are divided by the duration of the 2nd dendritic calcium transients: 0–8 s (**c**) and > 8 s (d) respectively (0–8 s, 139 spine calcium transients; > 8 s, 31 spine calcium transients). Data are mean ± s.e.m. *P < 0.05, **P < 0.01. n.s., not significant

Previous studies have shown that the level of dendritic calcium and the relative time windows between synaptic activity and dendritic calcium spikes are important for the induction of synapse potentiation or depotentiation [43, 55, 66–70]. To investigate whether and how abnormal dendritic calcium transients might affect synaptic activity and strength of L2/3 pyramidal neurons, we divided dendritic calcium transients into two groups based on their durations (0 – 8 s and longer than 8 s) (Fig. 5a). We then analyzed the peak ΔF/F_0_ of spine calcium transient before and after dendritic calcium transients in mice subjected to treadmill running (Fig. 5b). We found no significant difference in the peak ΔF/F_0_ of spine calcium transients before and after dendritic calcium transients with 0 – 8 s durations in either WT mice (Fig. 5c; *n* = 79 spine calcium transients from 4 mice. *P* = 0.135) or AD mice (Fig. 5e; *n* = 139 spine calcium transients from 5 mice. *P* = 0.858). Furthermore, we found a slight but not significant reduction in the peak amplitudes of spine calcium after long-duration (> 8 s) dendritic calcium transients in WT mice (Fig. 5d; *n* = 8 spine calcium transients from 4 mice. *P* = 0.076). Importantly, the peak ΔF/F_0_ of spine calcium transients was significantly reduced after the occurrence of long-duration (> 8 s) dendritic calcium transients in AD mice (Fig. 5f; *n* = 31 spine calcium transients from 5 mice. *P* = 0.005). Taken together, these findings suggest that prolonged dendritic calcium transients lead to a significant depotentiation in the peak ΔF/F_0_ of spine calcium activity in AD mice.

### The size of dendritic spines associated with long-duration dendritic calcium transients is reduced in AD mice

**Fig. 6:**
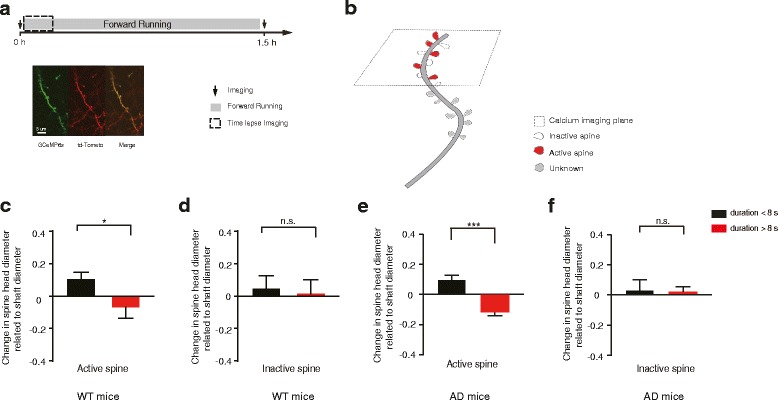
Dendritic calcium transients with abnormal long-durations reduce dendritic spine size in APP/PS1 mice. **a**. Schematic of experimental design and imaging spine structure and calcium transients. Top, time course of imaging and treadmill running. Bottom, images of dendrites labeled with GCaMP6s (green) and Td-tomato (red). **b**. Schematic showing inactive (blank) and active (red) spines on the calcium imaging plane. **c-d**. Size change of active (**c**) and inactive (**d**) spines on dendrites with short and prolonged dendritic calcium transients in WT mice (active spine number: duration <8 s, *n* = 33 and duration >8 s, *n* = 10; inactive spine number: duration <8 s, *n* = 9 and duration >8 s, *n* = 5. Wilcoxon signed rank test). **e**-**f**. Size change of active (**e**) and inactive (**f**) spines on dendrites with short and prolonged dendritic calcium transients in APP/PS1 mice (active spine number: duration <8 s, *n* = 30 and duration >8 s, *n* = 13; inactive spine number: duration <8 s, *n* = 15 and duration >8 s, *n* = 11. Wilcoxon signed rank test). Data are mean ± s.e.m. ***P < 0.001. n.s., not significant

To further investigate the impact of long-duration dendritic calcium transients in AD mice, we examined changes in dendritic spine number and size on apical tuft dendrites of L2/3 pyramidal neurons expressing both GCaMP6s and a structural maker, tdTomato, in the M1 region (Fig. 6a). In this experiment, we first imaged dendritic spines at 0 h, trained mice on the treadmill for ~1.5 h, and performed imaging again at 1.5 h. Over the period of 1.5 h running, we found no formation or elimination of dendritic spines in both WT and AD mice (Additional file 3: Figure S3: 72 and 89 spines examined in WT and AD mice, respectively).

Next, we examined whether long-duration dendritic calcium transients may affect spine size by comparing the size of spines before and after 1.5 h treadmill running. Previous studies of layer 5 pyramidal neurons have shown that dendritic spines active at dendritic spike generation are potentiated [43]. Furthermore, the ratio of GCaMP6 fluorescence intensity in spine head to neighboring shaft for active spines was 1.56 ± 0.13 and for inactive spines was 0.46 ± 0.02 [43]. Based on similar criteria (see also Methods), we classified active and non-active spines of layer 2/3 pyramidal neurons according to the ratio of GCaMP6 fluorescence intensity between spine heads and adjacent dendritic shafts at the time of dendritic calcium transient generation. We found that active spines showed an increase in size on dendrites with short-duration (< 8 s) calcium transients, but a reduction in size on dendrites with long-duration (> 8 s) calcium transients over 1.5 h in both WT mice (Fig. 6b, c; *n* = 33 spines from 4 mice, duration <8 s; *n* = 10 spines, duration >8 s; *P* = 0.04) and AD mice (Fig. 6b, e; *n* = 30 spines from 4 mice, duration <8 s; *n* = 13 spines, duration >8 s; *P* < 0.001). Furthermore, spines that were not active during dendritic calcium transients did not exhibit significant changes over 1.5 h, regardless of the durations of dendritic calcium transients in WT mice (Fig. 6b, d; *n* = 9 spines, duration <8 s; *n* = 5 spines, duration >8 s. *P* = 0.792) and in AD mice (Fig. 6b, f; *n* = 15 spines, duration <8 s; *n* = 11 spines, duration >8 s. *P* = 0.899). Taken together, these results indicate that dendritic spines active on dendritic branches with frequently-occurring, long-duration dendritic calcium transients are reduced in size in AD mice. Because dendritic spine size strongly correlates with synaptic strength, these findings also suggest that prolonged dendritic calcium transients observed in AD mice lead to abnormal reduction in synaptic strength in the motor cortex.

## Discussion

In this study, we report that before amyloid plaque deposition, dendritic calcium transients with abnormally long durations and high peak amplitudes occur on apical dendrites of layer 2/3 pyramidal neurons in the motor cortex of 3 month-old APPswe/PS1dE9 transgenic mice. These abnormally long dendritic calcium transients could be induced after administration of soluble Aβ oligomers. Furthermore, long-duration dendritic calcium transients are associated with the reduction of dendritic spine calcium peak amplitude and spine size. Together, these results show that prolonged dendritic calcium transients and increased synaptic depotentiation are early neuronal deficits that occur before amyloid plaque formation in AD mice.

Previous studies have shown that L2/3 cortical neurons exhibit both hyperactivity and hypoactivity in aged APP23 × PS45 mice [20]. These hyperactive neurons are mostly clustered around amyloid plaques, suggesting that the microenvironment near amyloid plaques play an important role in triggering hyperactivity of nearby neurons [20]. Our findings indicate that prior to amyloid plaque deposition, calcium activities of L2/3 neuronal somas during the quiet resting state are lower in 3-month-old APPswe/PS1dE9 AD mice than in WT mice. Furthermore, somatic calcium activities of these cells during treadmill running have no significant difference between AD and age-matched WT mice. Thus, unlike the hyperactivity of cortical neurons near amyloid plaques in aged APP23 × PS45 mice, there is no overall increase in the activities of cortical neurons in the plaque-free motor cortex of 3-month-old APPswe/PS1dE9 mice. It is worth to note that neuronal hyperactivity has been observed in the hippocampus of 1.5 month APP23 × PS45 mice before the formation of amyloid plaques [19]. Further studies are needed to investigate whether neuronal hyperactivity may occur in the hippocampus, but not in the motor cortex, of 3-month-old APPswe/PS1dE9 mice. If so, it would suggest that alterations of neuronal activities in AD mice occur at different stages in different brain regions.

One key finding of our study is that during the active running state, a fraction of apical dendrites of L2/3 pyramidal neurons exhibit dendritic calcium transients with abnormal long-duration and high-amplitude in the motor cortex of APPswe/PS1dE9 mice, prior to amyloid plaque formation. Recent studies have shown that dendritic calcium spikes are critical for changes of spine calcium activity and spine size in the motor cortex [43]. Consistent with the fundamental role of dendritic calcium elevation in synaptic plasticity, we found that peak amplitudes of dendritic spines decrease after prolonged dendritic calcium transients (> 8 s). Furthermore, long-duration dendritic calcium transients are associated with a reduction in the size of active spines, but not inactive spines, at the generation of dendritic calcium transients. Because calcium activities of neuronal somas were comparable between APPswe/PS1dE9 and WT mice during running, these results suggest the dysregulation of dendritic calcium and spine plasticity are among the earliest-occurring neuronal deficits (prior to the neuronal somatic hyperactivity and plaque formation) in the pathogenesis of AD.

We found that administration of Aβ oligomers in WT mice causes abnormal long-duration dendritic calcium transients. This finding is consistent with the detrimental roles of soluble Aβ oligomers in disrupting calcium homeostasis and synaptic plasticity in AD. Aβ oligomers and PS1 mutations can disrupt calcium release from ER [71–77]. Soluble Aβ oligomers can also disrupt dendritic calcium by interacting with NMDA receptors, modulating glutamate uptake and regulating calcium release from ER [35–41]. Such alterations of dendritic and spine calcium activity would likely have detrimental impacts on synaptic function and may lead to a progressive loss of synapses in AD. It is important to note that although we found synaptic depotentiation associated with prolonged dendritic calcium activity, we did not observe an increase of spine loss over 1.5 h of treadmill running. Future studies are therefore needed to investigate the relationship among long-duration dendritic calcium activity, spine depotentiation and potential spine elimination over extended periods of time to better understand synapse loss in AD pathogenesis.

Many lines of evidence indicate that NMDAR activation plays important roles in the generation of dendritic calcium spikes and synaptic plasticity [43, 58–60]. Consistent with these previous studies [43, 58–60], we observed that NMDAR antagonist MK801 application significantly reduced the frequency, duration and peak amplitude of dendritic and spine calcium transients (Additional file 4: Figure S4 a-f). Furthermore, after MK801 application, there were no significant changes in the peak amplitude of spine calcium transients before and after dendritic calcium transients (Additional file 4: Figure S4 g). These findings raise the possibility that the use of NMDAR antagonists such as memantine may slow down AD progression in part by reducing prolonged dendritic calcium activity and synaptic abnormalities. Future studies are needed to investigate whether memantine and other pharmacological agents targeting abnormal dendritic calcium activities and their signaling may help the treatment of AD by alleviating synaptic depotentiation and loss.

## Conclusion

Our findings show that abnormal long-duration dendritic calcium transients and decreases in spine size occur prior to amyloid plaque formation and overall changes of neuronal somatic activity in the APPswe/PS1dE9 mouse model of AD. The abnormal long-duration and high-amplitude dendritic calcium transients can be induced by soluble Aβ oligomers and may contribute to synaptic deficits during the early pathogenesis of AD.

## Additional files

Additional file 1: Figure S1. Soluble Aβ oligomer injection induces higher neuronal calcium activity in virus injected WT mice during running. **a**. Distributions of the frequency, duration, peak ΔF/F_0_ and total integrated activity of somatic calcium transients in vehicle-injected mice and Aβ oligomer-injected mice during quiet resting (Vehicle: 4 mice, 80 somas; Aβ oligomer: 4 mice, 89 somas. Mann-Whitney U Test). **b**. Distributions of the frequency, duration, peak ΔF/F_0_ and total integrated activity of somatic calcium transients in vehicle-injected mice and Aβ oligomer-injected mice during running (Vehicle: 4 mice, 80 somas; Aβ oligomer: 4 mice, 89 somas. Mann-Whitney U Test). *P < 0.05, **P < 0.01. n.s., not significant. (PDF 389 kb)

Additional file 2: Figure S2. The overall spine calcium activities are comparable between vehicle and soluble Aβ oligomer-injected mice. **a**. Distributions of the frequency, duration, peak ΔF/F_0_ and total integrated activity of spine calcium transients in vehicle and Aβ oligomer-injected mice during quiet resting state (Vehicle: 4 mice, 53 spines; Aβ oligomer: 4 mice, 62 spines. Mann-Whitney U Test). **b**. Distributions of the frequency, duration, peak ΔF/F_0_ and total integrated activity of spine calcium transients in vehicle and Aβ oligomer-injected mice during running (Vehicle: 4 mice, 66 spines; Aβ oligomer: 4 mice, 56 spines. Mann-Whitney U Test). *P < 0.05. n.s., not significant. (PDF 374 kb)

Additional file 3: Figure S3. Spine turnover rate is comparable in WT and AD mice during 1.5 h treadmill running. Spine turnover rate (stable, elimination and formation rate) in WT mice (*n* = 4) and AD mice (*n* = 4). No spine formation or elimination was found over a 1.5 h period of treadmill running. (PDF 258 kb)

Additional file 4: Figure S4. The dendritic calcium activities decrease after MK801 application during running. **a-c**. The frequency (a), duration (b) and peak amplitude (c) of dendrite calcium transients before and after MK801 application (10 min). (60 dendrites from 4 mice, Student’s T test). **d-f**. The frequency (d), duration (e) and peak amplitude (f) of spine calcium transients before and after MK801 application (10 min). (58 spines from 4 mice, Student’s T test). **g**. No changes of spine peak ΔF/F_0_ before and after dendritic calcium transients after MK801 application (26 spines from 4 mice, Student’s T test). Data are mean ± s.e.m. ***P < 0.001. n.s., not significant. (PDF 330 kb)

## Abbreviations

AD: Alzheimer’s disease
APP/PS1: APPswe/PSEN1dE9
Aβ: β-amyloid

## References

[1] Terry RD, Masliah E, Salmon DP, Butters N, DeTeresa R, Hill R, Hansen LA, Katzman R (1991). Physical basis of cognitive alterations in Alzheimer's disease: synapse loss is the major correlate of cognitive impairment. Ann Neurol.

[2] Masliah E, Crews L, Hansen L (2006). Synaptic remodeling during aging and in Alzheimer's disease. J Alzheimers Dis.

[3] Scheff SW, Price DA (1993). Synapse loss in the temporal lobe in Alzheimer's disease. Ann Neurol.

[4] Selkoe DJ (2002). Alzheimer's disease is a synaptic failure. Science.

[5] Scheff SW, Price DA, Schmitt FA, Mufson EJ (2006). Hippocampal synaptic loss in early Alzheimer's disease and mild cognitive impairment. Neurobiol Aging.

[6] Scheff SW, Price DA, Schmitt FA, DeKosky ST, Mufson EJ (2007). Synaptic alterations in CA1 in mild Alzheimer disease and mild cognitive impairment. Neurology.

[7] Honer WG (2003). Pathology of presynaptic proteins in Alzheimer's disease: more than simple loss of terminals. Neurobiol Aging.

[8] Jacobsen JS, CC W, Redwine JM, Comery TA, Arias R, Bowlby M, Martone R, Morrison JH, Pangalos MN, Reinhart PH, Bloom FE (2006). Early-onset behavioral and synaptic deficits in a mouse model of Alzheimer's disease. Proc Natl Acad Sci U S A.

[9] CC W, Chawla F, Games D, Rydel RE, Freedman S, Schenk D, Young WG, Morrison JH, Bloom FE (2004). Selective vulnerability of dentate granule cells prior to amyloid deposition in PDAPP mice: digital morphometric analyses. Proc Natl Acad Sci U S A.

[10] Reddy PH, Mani G, Park BS, Jacques J, Murdoch G, Whetsell W, Kaye J, Manczak M (2005). Differential loss of synaptic proteins in Alzheimer's disease: implications for synaptic dysfunction. J Alzheimers Dis.

[11] Ingelsson M, Fukumoto H, Newell KL, Growdon JH, Hedley-Whyte ET, Frosch MP, Albert MS, Hyman BT, Irizarry MC (2004). Early Abeta accumulation and progressive synaptic loss, gliosis, and tangle formation in AD brain. Neurology.

[12] Maras PM, Molet J, Chen Y, Rice C, Ji SG, Solodkin A, Baram TZ (2014). Preferential loss of dorsal-hippocampus synapses underlies memory impairments provoked by short, multimodal stress. Mol Psychiatry.

[13] Vanleeuwen JE, Penzes P (2012). Long-term perturbation of spine plasticity results in distinct impairments of cognitive function. J Neurochem.

[14] Albers MW, Gilmore GC, Kaye J, Murphy C, Wingfield A, Bennett DA, Boxer AL, Buchman AS, Cruickshanks KJ, Devanand DP (2015). At the interface of sensory and motor dysfunctions and Alzheimer's disease. Alzheimers Dement.

[15] Chainay H, Louarn C, Humphreys GW (2006). Ideational action impairments in Alzheimer's disease. Brain Cogn.

[16] Hebert LE, Bienias JL, McCann JJ, Scherr PA, Wilson RS, Evans DA (2010). Upper and lower extremity motor performance and functional impairment in Alzheimer's disease. Am J Alzheimers Dis Other Demen.

[17] Minkeviciene R, Rheims S, Dobszay MB, Zilberter M, Hartikainen J, Fulop L, Penke B, Zilberter Y, Harkany T, Pitkanen A, Tanila H (2009). Amyloid beta-induced neuronal hyperexcitability triggers progressive epilepsy. J Neurosci.

[18] Quiroz YT, Budson AE, Celone K, Ruiz A, Newmark R, Castrillon G, Lopera F, Stern CE (2010). Hippocampal hyperactivation in presymptomatic familial Alzheimer's disease. Ann Neurol.

[19] Busche MA, Chen X, Henning HA, Reichwald J, Staufenbiel M, Sakmann B, Konnerth A (2012). Critical role of soluble amyloid-beta for early hippocampal hyperactivity in a mouse model of Alzheimer's disease. Proc Natl Acad Sci U S A.

[20] Busche MA, Eichhoff G, Adelsberger H, Abramowski D, Wiederhold KH, Haass C, Staufenbiel M, Konnerth A, Garaschuk O (2008). Clusters of hyperactive neurons near amyloid plaques in a mouse model of Alzheimer's disease. Science.

[21] Lefort S, Tomm C, Floyd Sarria JC, Petersen CC (2009). The excitatory neuronal network of the C2 barrel column in mouse primary somatosensory cortex. Neuron.

[22] Petreanu L, Mao T, Sternson SM, Svoboda K (2009). The subcellular organization of neocortical excitatory connections. Nature.

[23] Grutzendler J, Helmin K, Tsai J, Gan WB (2007). Various dendritic abnormalities are associated with fibrillar amyloid deposits in Alzheimer's disease. Ann N Y Acad Sci.

[24] Perez-Cruz C, Nolte MW, van Gaalen MM, Rustay NR, Termont A, Tanghe A, Kirchhoff F, Ebert U (2011). Reduced spine density in specific regions of CA1 pyramidal neurons in two transgenic mouse models of Alzheimer's disease. J Neurosci.

[25] del Valle J, Bayod S, Camins A, Beas-Zarate C, Velazquez-Zamora DA, Gonzalez-Burgos I, Pallas M (2012). Dendritic spine abnormalities in hippocampal CA1 pyramidal neurons underlying memory deficits in the SAMP8 mouse model of Alzheimer's disease. J Alzheimers Dis.

[26] Tsai J, Grutzendler J, Duff K, Gan WB (2004). Fibrillar amyloid deposition leads to local synaptic abnormalities and breakage of neuronal branches. Nat Neurosci.

[27] Palop JJ, Chin J, Roberson ED, Wang J, Thwin MT, Bien-Ly N, Yoo J, Ho KO, GQ Y, Kreitzer A (2007). Aberrant excitatory neuronal activity and compensatory remodeling of inhibitory hippocampal circuits in mouse models of Alzheimer's disease. Neuron.

[28] Koukouli F, Rooy M, Maskos U (2016). Early and progressive deficit of neuronal activity patterns in a model of local amyloid pathology in mouse prefrontal cortex. Aging (Albany NY).

[29] Lue LF, Kuo YM, Roher AE, Brachova L, Shen Y, Sue L, Beach T, Kurth JH, Rydel RE, Rogers J (1999). Soluble amyloid beta peptide concentration as a predictor of synaptic change in Alzheimer's disease. Am J Pathol.

[30] Naslund J, Haroutunian V, Mohs R, Davis KL, Davies P, Greengard P, Buxbaum JD (2000). Correlation between elevated levels of amyloid beta-peptide in the brain and cognitive decline. JAMA.

[31] Allsop D, Mayes J (2014). Amyloid beta-peptide and Alzheimer's disease. Essays Biochem.

[32] Moechars D, Dewachter I, Lorent K, Reverse D, Baekelandt V, Naidu A, Tesseur I, Spittaels K, Haute CV, Checler F (1999). Early phenotypic changes in transgenic mice that overexpress different mutants of amyloid precursor protein in brain. J Biol Chem.

[33] Mucke L, Masliah E, GQ Y, Mallory M, Rockenstein EM, Tatsuno G, Hu K, Kholodenko D, Johnson-Wood K, McConlogue L (2000). High-level neuronal expression of abeta 1-42 in wild-type human amyloid protein precursor transgenic mice: synaptotoxicity without plaque formation. J Neurosci.

[34] Busche MA, Kekus M, Adelsberger H, Noda T, Forstl H, Nelken I, Konnerth A (2015). Rescue of long-range circuit dysfunction in Alzheimer's disease models. Nat Neurosci.

[35] Texido L, Martin-Satue M, Alberdi E, Solsona C, Matute C (2011). Amyloid beta peptide oligomers directly activate NMDA receptors. Cell Calcium.

[36] Pellistri F, Bucciantini M, Relini A, Nosi D, Gliozzi A, Robello M, Stefani M (2008). Nonspecific interaction of prefibrillar amyloid aggregates with glutamatergic receptors results in Ca2+ increase in primary neuronal cells. J Biol Chem.

[37] Snyder EM, Nong Y, Almeida CG, Paul S, Moran T, Choi EY, Nairn AC, Salter MW, Lombroso PJ, Gouras GK, Greengard P (2005). Regulation of NMDA receptor trafficking by amyloid-beta. Nat Neurosci.

[38] Li S, Hong S, Shepardson NE, Walsh DM, Shankar GM, Selkoe D (2009). Soluble oligomers of amyloid Beta protein facilitate hippocampal long-term depression by disrupting neuronal glutamate uptake. Neuron.

[39] Green KN, Demuro A, Akbari Y, Hitt BD, Smith IF, Parker I, LaFerla FM (2008). SERCA pump activity is physiologically regulated by presenilin and regulates amyloid beta production. J Cell Biol.

[40] Cheung KH, Shineman D, Muller M, Cardenas C, Mei L, Yang J, Tomita T, Iwatsubo T, Lee VM, Foskett JK (2008). Mechanism of Ca2+ disruption in Alzheimer's disease by presenilin regulation of InsP3 receptor channel gating. Neuron.

[41] Christensen RA, Shtifman A, Allen PD, Lopez JR, Querfurth HW (2004). Calcium dyshomeostasis in beta-amyloid and tau-bearing skeletal myotubes. J Biol Chem.

[42] Chen TW, Wardill TJ, Sun Y, Pulver SR, Renninger SL, Baohan A, Schreiter ER, Kerr RA, Orger MB, Jayaraman V (2013). Ultrasensitive fluorescent proteins for imaging neuronal activity. Nature.

[43] Cichon J, Gan WB (2015). Branch-specific dendritic ca(2+) spikes cause persistent synaptic plasticity. Nature.

[44] Jankowsky JL, Slunt HH, Ratovitski T, Jenkins NA, Copeland NG, Borchelt DR (2001). Co-expression of multiple transgenes in mouse CNS: a comparison of strategies. Biomol Eng.

[45] Borchelt DR, Davis J, Fischer M, Lee MK, Slunt HH, Ratovitsky T, Regard J, Copeland NG, Jenkins NA, Sisodia SS, Price DL (1996). A vector for expressing foreign genes in the brains and hearts of transgenic mice. Genet Anal.

[46] Garcia-Alloza M, Robbins EM, Zhang-Nunes SX, Purcell SM, Betensky RA, Raju S, Prada C, Greenberg SM, Bacskai BJ, Frosch MP (2006). Characterization of amyloid deposition in the APPswe/PS1dE9 mouse model of Alzheimer disease. Neurobiol Dis.

[47] Heiss JK, Barrett J, Yu Z, Haas LT, Kostylev MA, Strittmatter SM (2017). Early activation of experience-independent dendritic spine turnover in a mouse model of Alzheimer's disease. Cereb Cortex.

[48] Zhao YJ, Sivaji S, Chiang MC, Ali H, Zukowski M, Ali D, Kennedy B, Sklyar A, Cheng A, Guo ZH (2017). Amyloid Beta peptides block new synapse assembly by Nogo receptor-mediated inhibition of T-type calcium channels. Neuron.

[49] Li W, Ma L, Yang G, Gan WB (2017). REM sleep selectively prunes and maintains new synapses in development and learning. Nat Neurosci.

[50] Yang G, Lai CSW, Cichon J, Ma L, Li W, Gan WB (2014). Sleep promotes branch-specific formation of dendritic spines after learning. Science.

[51] Li CX, Waters RS (1991). Organization of the mouse motor cortex studied by retrograde tracing and intracortical microstimulation (ICMS) mapping. Can J Neurol Sci.

[52] Helmchen F, Imoto K, Sakmann B (1996). Ca2+ buffering and action potential-evoked Ca2+ signaling in dendrites of pyramidal neurons. Biophys J.

[53] Svoboda K, Helmchen F, Denk W, Tank DW (1999). Spread of dendritic excitation in layer 2/3 pyramidal neurons in rat barrel cortex in vivo. Nat Neurosci.

[54] Myoga MH, Beierlein M, Regehr WG (2009). Somatic spikes regulate dendritic signaling in small neurons in the absence of backpropagating action potentials. J Neurosci.

[55] Golding NL, Staff NP, Spruston N (2002). Dendritic spikes as a mechanism for cooperative long-term potentiation. Nature.

[56] Humeau Y, Luthi A (2007). Dendritic calcium spikes induce bi-directional synaptic plasticity in the lateral amygdala. Neuropharmacology.

[57] Waters J, Helmchen F (2004). Boosting of action potential backpropagation by neocortical network activity in vivo. J Neurosci.

[58] NL X, Harnett MT, Williams SR, Huber D, O'Connor DH, Svoboda K, Magee JC (2012). Nonlinear dendritic integration of sensory and motor input during an active sensing task. Nature.

[59] Grienberger C, Chen X, Konnerth A (2014). NMDA receptor-dependent multidendrite ca(2+) spikes required for hippocampal burst firing in vivo. Neuron.

[60] Palmer LM, Shai AS, Reeve JE, Anderson HL, Paulsen O, Larkum ME (2014). NMDA spikes enhance action potential generation during sensory input. Nat Neurosci.

[61] Lesne S, Koh MT, Kotilinek L, Kayed R, Glabe CG, Yang A, Gallagher M, Ashe KH (2006). A specific amyloid-beta protein assembly in the brain impairs memory. Nature.

[62] Shankar GM, Li S, Mehta TH, Garcia-Munoz A, Shepardson NE, Smith I, Brett FM, Farrell MA, Rowan MJ, Lemere CA (2008). Amyloid-beta protein dimers isolated directly from Alzheimer's brains impair synaptic plasticity and memory. Nat Med.

[63] Woods NK, Padmanabhan J (2012). Neuronal calcium signaling and Alzheimer's disease. Adv Exp Med Biol.

[64] Arbel-Ornath M, Hudry E, Boivin JR, Hashimoto T, Takeda S, Kuchibhotla KV, Hou S, Lattarulo CR, Belcher AM, Shakerdge N (2017). Soluble oligomeric amyloid-beta induces calcium dyshomeostasis that precedes synapse loss in the living mouse brain. Mol Neurodegener.

[65] Dinamarca MC, Colombres M, Cerpa W, Bonansco C, Inestrosa NC (2008). Beta-amyloid oligomers affect the structure and function of the postsynaptic region: role of the Wnt signaling pathway. Neurodegener Dis.

[66] Holthoff K, Kovalchuk Y, Yuste R, Konnerth A (2004). Single-shock LTD by local dendritic spikes in pyramidal neurons of mouse visual cortex. J Physiol.

[67] Kampa BM, Letzkus JJ, Stuart GJ (2006). Requirement of dendritic calcium spikes for induction of spike-timing-dependent synaptic plasticity. J Physiol.

[68] Lisman J, Spruston N (2005). Postsynaptic depolarization requirements for LTP and LTD: a critique of spike timing-dependent plasticity. Nat Neurosci.

[69] Nevian T, Sakmann B (2004). Single spine Ca2+ signals evoked by coincident EPSPs and backpropagating action potentials in spiny stellate cells of layer 4 in the juvenile rat somatosensory barrel cortex. J Neurosci.

[70] Sheffield ME, Dombeck DA (2015). Calcium transient prevalence across the dendritic arbour predicts place field properties. Nature.

[71] Berridge MJ (2013). Dysregulation of neural calcium signaling in Alzheimer disease, bipolar disorder and schizophrenia. Prion.

[72] Zeiger W, Vetrivel KS, Buggia-Prevot V, Nguyen PD, Wagner SL, Villereal ML, Thinakaran G (2013). Ca2+ influx through store-operated Ca2+ channels reduces Alzheimer disease beta-amyloid peptide secretion. J Biol Chem.

[73] Supnet C, Bezprozvanny I (2010). The dysregulation of intracellular calcium in Alzheimer disease. Cell Calcium.

[74] Jensen LE, Bultynck G, Luyten T, Amijee H, Bootman MD, Roderick HL (2013). Alzheimer's disease-associated peptide Abeta42 mobilizes ER ca(2+) via InsP3R-dependent and -independent mechanisms. Front Mol Neurosci.

[75] Mattson MP. ER calcium and Alzheimer's disease: in a state of flux. Science signaling 2010;3:pe10. 10.1126/scisignal.3114pe10 PMC3091478 20332425

[76] Small DH (2009). Dysregulation of calcium homeostasis in Alzheimer's disease. Neurochem Res.

[77] Popugaeva E, Bezprozvanny I (2013). Role of endoplasmic reticulum Ca2+ signaling in the pathogenesis of Alzheimer disease. Front Mol Neurosci.

