# Supplementary figures and images for: Abnormal dendritic calcium activity and synaptic depotentiation occur early in a mouse model of Alzheimer’s disease
Source: Mol Neurodegener. 2017 Nov 14;12:86. doi: 10.1186/s13024-017-0228-2 (PMC5686812; doi:10.1186/s13024-017-0228-2)

a

Quiet resting

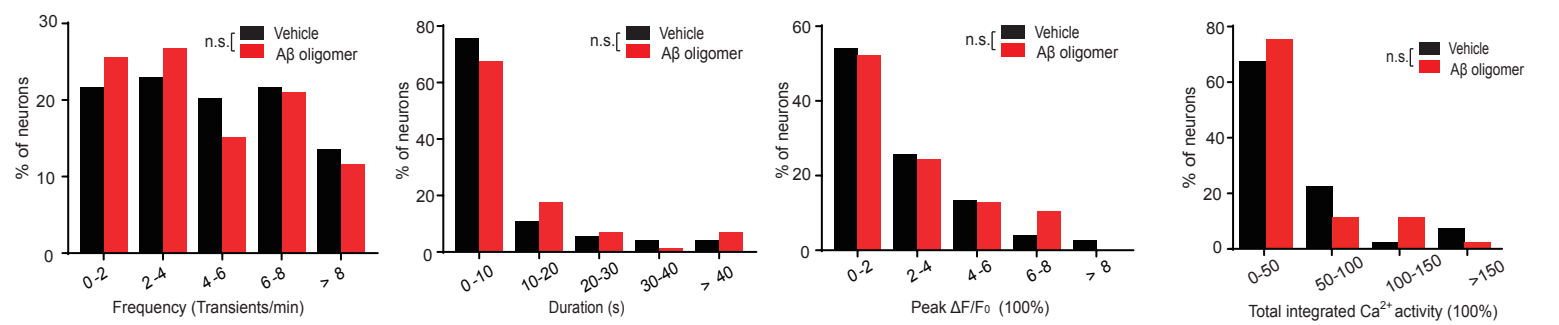

b

Running

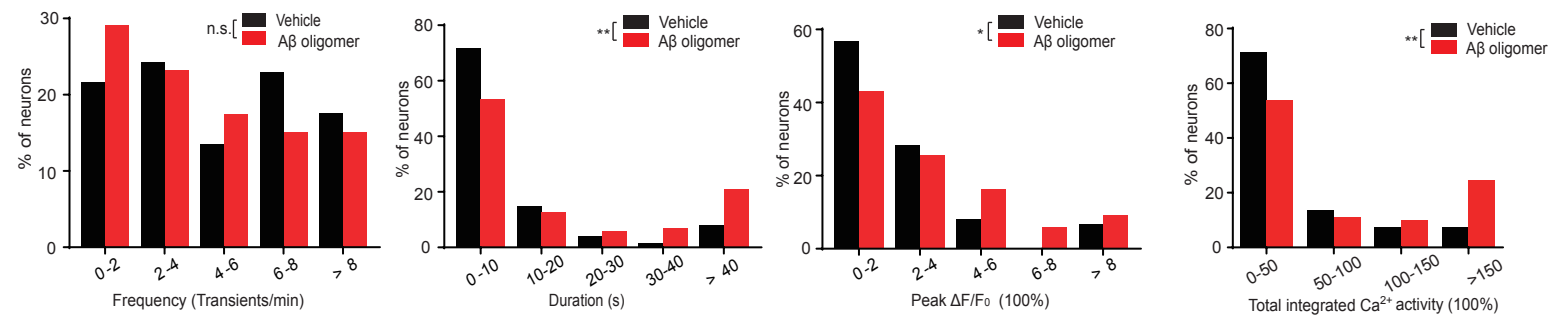

Supplement: Supplementary file 1 — Soluble Aβ oligomer injection induces higher neuronal calcium activity in virus injected WT mice during running. a. Distributions of the frequency, duration, peak ΔF/F0 and total integrated activity of somatic calcium transients in vehicle-injected mice and Aβ oligomer-injected mice during quiet resting (Vehicle: 4 mice, 80 somas; Aβ oligomer: 4 mice, 89 somas. Mann-Whitney U Test). b. Distributions of the frequency, duration, peak ΔF/F0 and total integrated activity of somatic calcium transients in vehicle-injected mice and Aβ oligomer-injected mice during running (Vehicle: 4 mice, 80 somas; Aβ oligomer: 4 mice, 89 somas. Mann-Whitney U Test). *P < 0.05, **P < 0.01. n.s., not significant. (PDF 389 kb) [file 13024_2017_228_MOESM1_ESM.pdf]

**a**

Quiet resting

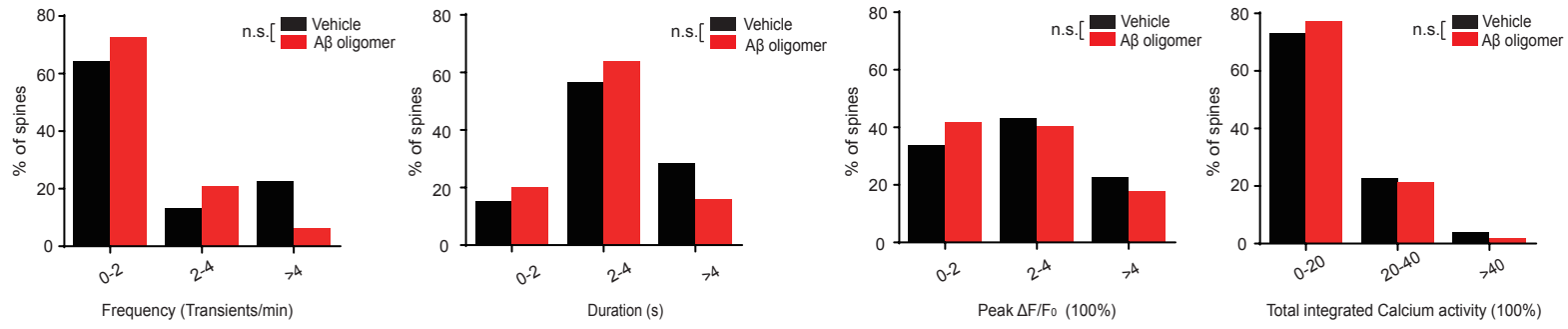**b**

Running

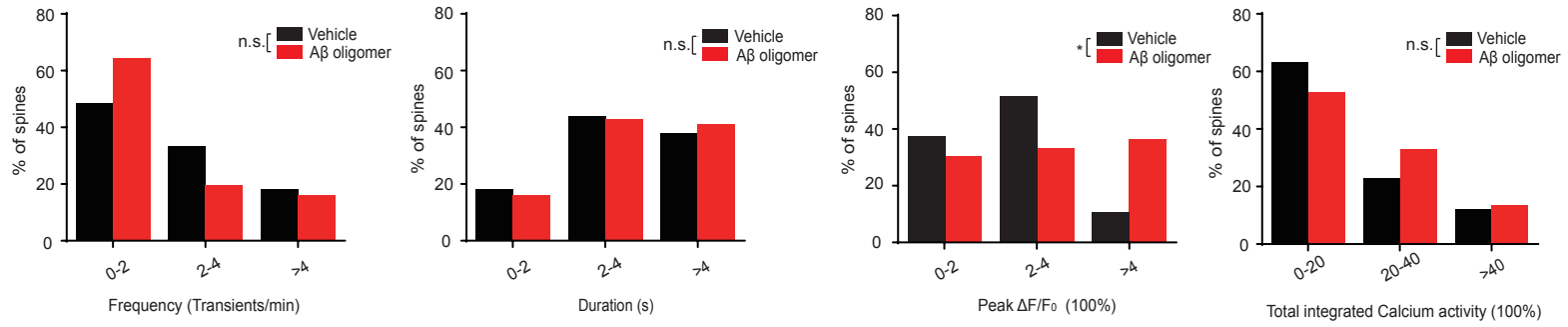

Supplement: Supplementary file 2 — The overall spine calcium activities are comparable between vehicle and soluble Aβ oligomer-injected mice. a. Distributions of the frequency, duration, peak ΔF/F0 and total integrated activity of spine calcium transients in vehicle and Aβ oligomer-injected mice during quiet resting state (Vehicle: 4 mice, 53 spines; Aβ oligomer: 4 mice, 62 spines. Mann-Whitney U Test). b. Distributions of the frequency, duration, peak ΔF/F0 and total integrated activity of spine calcium transients in vehicle and Aβ oligomer-injected mice during running (Vehicle: 4 mice, 66 spines; Aβ oligomer: 4 mice, 56 spines. Mann-Whitney U Test). *P < 0.05. n.s., not significant. (PDF 374 kb) [file 13024_2017_228_MOESM2_ESM.pdf]

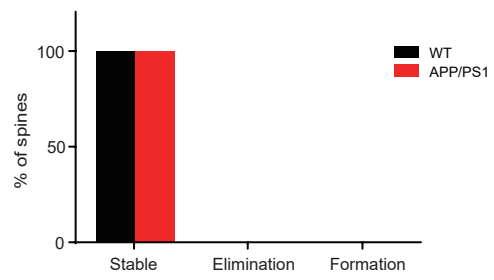

Supplement: Supplementary file 3 — Spine turnover rate is comparable in WT and AD mice during 1.5 h treadmill running. Spine turnover rate (stable, elimination and formation rate) in WT mice (n = 4) and AD mice (n = 4). No spine formation or elimination was found over a 1.5 h period of treadmill running. (PDF 258 kb) [file 13024_2017_228_MOESM3_ESM.pdf]

Dendritic calcium activity

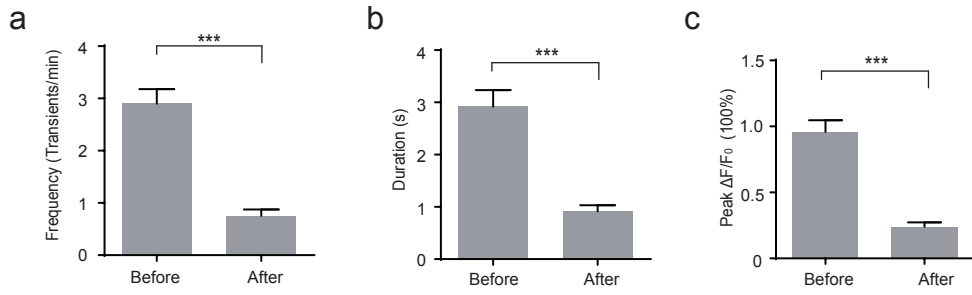

Spine calcium activity

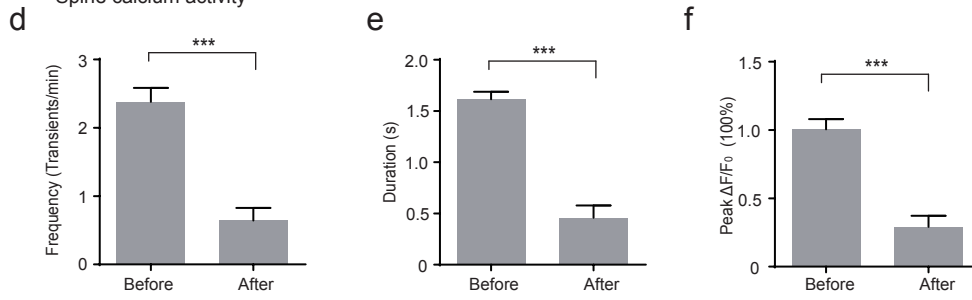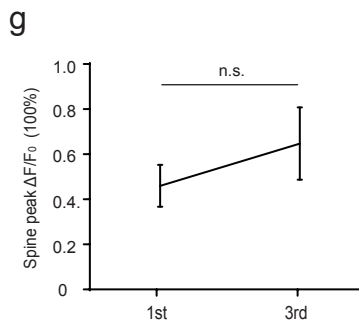

Supplement: Supplementary file 4 — The dendritic calcium activities decrease after MK801 application during running. a-c. The frequency (a), duration (b) and peak amplitude (c) of dendrite calcium transients before and after MK801 application (10 min). (60 dendrites from 4 mice, Student’s T test). d-f. The frequency (d), duration (e) and peak amplitude (f) of spine calcium transients before and after MK801 application (10 min). (58 spines from 4 mice, Student’s T test). g. No changes of spine peak ΔF/F0 before and after dendritic calcium transients after MK801 application (26 spines from 4 mice, Student’s T test). Data are mean ± s.e.m. ***P < 0.001. n.s., not significant. (PDF 330 kb) [file 13024_2017_228_MOESM4_ESM.pdf]
